# Horizontal Transfer of a Nitrate Assimilation Gene Cluster and Ecological Transitions in Fungi: A Phylogenetic Study

**DOI:** 10.1371/journal.pone.0001097

**Published:** 2007-10-31

**Authors:** Jason C. Slot, David S. Hibbett

**Affiliations:** Department of Biology, Clark University, Worcester, Massachusetts, United States of America; University of Queensland, Australia

## Abstract

High affinity nitrate assimilation genes in fungi occur in a cluster (fHANT-AC) that can be coordinately regulated. The clustered genes include *nrt2*, which codes for a high affinity nitrate transporter; *euknr*, which codes for nitrate reductase; and *NAD(P)H-nir*, which codes for nitrite reductase. Homologs of genes in the fHANT-AC occur in other eukaryotes and prokaryotes, but they have only been found clustered in the oomycete *Phytophthora* (heterokonts). We performed independent and concatenated phylogenetic analyses of homologs of all three genes in the fHANT-AC. Phylogenetic analyses limited to fungal sequences suggest that the fHANT-AC has been transferred horizontally from a basidiomycete (mushrooms and smuts) to an ancestor of the ascomycetous mold *Trichoderma reesei*. Phylogenetic analyses of sequences from diverse eukaryotes and eubacteria, and cluster structure, are consistent with a hypothesis that the fHANT-AC was assembled in a lineage leading to the oomycetes and was subsequently transferred to the Dikarya (Ascomycota+Basidiomycota), which is a derived fungal clade that includes the vast majority of terrestrial fungi. We propose that the acquisition of high affinity nitrate assimilation contributed to the success of Dikarya on land by allowing exploitation of nitrate in aerobic soils, and the subsequent transfer of a complete assimilation cluster improved the fitness of *T. reesei* in a new niche. Horizontal transmission of this cluster of functionally integrated genes supports the “selfish operon” hypothesis for maintenance of gene clusters.

## Introduction

A cluster of nitrate assimilation genes occurs in the ascomycetes *Aspergillus nidulans* [1] and *Pichia angusta* [2] and in the basidiomycetes *Hebeloma cylindrosporum*, which is mycorrhizal, and *Phanerochaete chrysosporium* [3], which is a wood-decayer. This cluster, abbreviated fHANT-AC, encodes a high affinity nitrate transporter (NRT2, TCDB 2.A.1.8.5) along with a nitrate reductase (EUKNR, EC1.7.1.3) and a ferredoxin-independent assimilatory nitrite reductase (NAD[P]H-NIR, EC1.7.1.4). The latter two proteins are required for the reduction of nitrate to ammonium [4]. Ascomycetes and basidiomycetes are sister taxa that together form a clade called Dikarya, which contains the vast majority of described species of filamentous, terrestrial fungi. Without regard to clustering, *nrt2* is widely distributed in bacteria, plants, heterokonts and other groups, but not in opisthokonts outside Dikarya [5]. *Euknr* is restricted to eukaryotes, and absent from opisthokonts outside Dikarya, and *NAD(P)H-nir* is known to occur in Dikarya, heterokonts and bacteria.

The clustering of functionally related genes may be selectively favorable because it facilitates coordinated expression of the constituent genes [6]. The “selfish operon” hypothesis puts forth that clusters can result from the selective maintenance of fitness-improving genes following horizontal gene transfer (HGT) events [7]. The clustering of genes in selfish operons greatly increases success of HGT events when a phenotypic advantage or ability to exploit a new niche is conferred to the recipient [8]. Gene clusters may facilitate the acquisition of entire metabolic pathways in the fungi [9], [10]. Examples mainly involve secondary metabolite clusters, which do not necessarily require HGT between fungal species to explain their distribution [11], and pathogenicity gene clusters [9], [12]. Transfers of supernumerary chromosomes and large fragments of chromatin remain the most compelling mechanisms [12], [13], [14] for the transfer of metabolic pathways.

After discovering a potential horizontal transfer of *nrt2* previously [5], we conducted a series of phylogenetic analyses to reconstruct the history of the fHANT-AC and its constituent genes. We first searched available eukaryotic genome sequences for homologs of genes in fHANT-AC. We then estimated the organismal phylogeny of ascomycetes, basidiomycetes, and oomycetes using rRNA and RPB2 gene sequences. We contrasted the organismal phylogeny with results of independent and concatenated phylogenetic analyses of genes in the fHANT-AC, which we performed at two phylogenetic scales: within the fungi (rooted with the heterokont *Phytophthora*), and across diverse eukaryotes and eubacteria (rooted with selected prokaryotes). Phylogenetic analyses within the fungi suggest that the nitrate assimilation cluster in the ascomycete *T. reesei* was obtained by HGT from a basidiomycete and corresponds to a change in nutritional mode. This is the best evidence to date that horizontal transfer of a metabolic pathway can facilitate niche shift in fungi. Less conclusive phylogenetic and structural evidence from analyses across diverse eukaryotes and eubacteria cannot reject a heterokont origin of the nitrate assimilation cluster in Dikarya.

## Results

### Distribution of Genes of the fHANT-AC in Eukaryotes is Patchy

Homologs of each of the three genes of the fHANT-AC (SI Table S1) are limited to Viridiplantae, Rhodophyta, heterokonts (oomycetes+diatoms in this dataset) and Dikarya, with one exception in *Nematostella vectensis* (Metazoa), which possesses bacteria-like *nrt2* and *nitrite reductase* homologs (http://genome.jgi-psf.org/Nemve1/Nemve1.home.html). Viridiplantae and Rhodophyta possess genes homologous to the dikarya/heterokont *nrt2* and *euknr*, but not the fungal/heterokont *NAD(P)H-nir*. Instead, these groups maintain a ferredoxin-dependent nitrite reductase gene (*cpnir*, EC1.7.7.1) of probable plastid/cyanobacterial origin [15]. Diatoms possess the three Dikarya/oomycete homologs in addition to *cpnir* (http://genome.jgi-psf.org/Thaps3/Thaps3.home.html). Diverse eubacteria possess *nrt2* and *NAD(P)H-nir* homologs, but not *euknr* which has an exclusively eukaryotic distribution [16]. From the *Populus trichocarpa* genome, we recovered a β proteobacteria-like *NAD(P)H-nir*. Homologs of the dikarya/oomycete genes are absent from most Dikarya yeasts (with the exception of *Pichia angusta*), filamentous fungi outside Dikarya (*Rhizopus oryzae* and *Phycomyces blakesleeanus*), and the chytrid *Batrachochytrium dendrobatis*.

### Phylogenetic Analyses Suggest Horizontal Gene Transfer of the fHANT-AC

**Figure 1:**
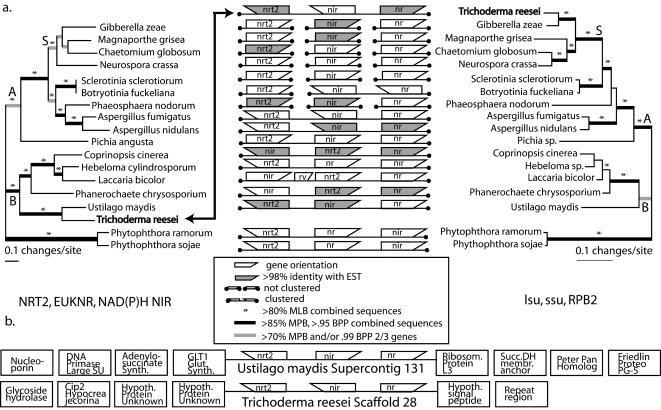
Phylogeny and clustering of the fHANT-AC. (a) Organismal phylogeny based on rRNA and RPB2 genes (right), and gene phylogeny of the fHANT-AC cluster (left), along with data on internal gene order and extent of clustering (center). Phylograms are from 50% majority rule Bayesian consensus. Shading of genes indicates confirmed expression of sequences in GenBank (dbEST:CF878787.1, CB906451.1, CF872359, CF865713, CB895628). Thick black branches denote strong support for the complete combined sequence (Bayesian posterior probabilities [BPP]>0.95, maximum parsimony bootstrap percentages [MPB]>85), and thick gray branches denote strong support from two of three individual genes (BPP 0.99 and/or MPB 70%). Strong support from maximum likelihood bootstraps ([MLB]>80%) is indicated by an *. Actual support values for critical nodes are in Table 1. Clades labeled A (Ascomycota), B (Basidiomycota) and S (Sordariomycetes) follow James *et al.* (2006). Contributing phylogenetic analyses are in SI Fig. S1a,b. (b) Description of open reading frames flanking the fHANT-AC in *T. reesei* and *U. maydis*. Complete descriptions are available at genome project websites (see SI Table S1).

A comparison of the organismal phylogeny and the phylogeny of fHANT-AC within fungi provides support for the hypothesis that fHANT-AC has been transferred horizontally from basidiomycetes to an ascomycete (Fig. 1a). In separate and combined analyses of nuclear ribosomal RNA genes (rDNA) and translated *rpb2* (RPB2) sequences using Bayesian, maximum likelihood (ML) and maximum parsimony (MP) methods (BPP = Bayesian Posterior Probabilities, MLB = Maximum Likelihood Bootstrap, MPB = Maximum Parsimony Bootstrap), we recovered species relationships that are congruent with previous molecular phylogenies [17]. We received strong support (1.0 BPP, 100 MPB combined analysis and 100MLB rDNA and RPB2 analyses unless otherwise noted) for the monophyly of Basidiomycota (RPB2 = 97 MLB, rDNA = 63 MLB) including *Ustilago maydis*, the ascomycetes (rDNA = 81 MLB), Pezizomycotina (non-yeast ascomycete lineages), and Sordariomycetes including *Trichoderma reesei*.

**Table 1:** Alignments including fungal and *Phythophthora* sequences.

Dataset			Clade			
	**Fungi**	**Ascomycetes**	**Pezizomycetes**	***T. reesei*** **+Basidiomycetes**	**Sordariomycetes**	***Ustilago maydis*** **+** ***T. reesei***
**NRT2**	1.0B 100L 100P1	-	-	NS2B NSL NSP	-	1.0B 100L 99P
**EUKNR**	1.0B 100L 100P	-	-	1.0B 99L NSP	-	1.0B 100L 100P
**NAD(P)H NIR**	1.0B 100L 100P	-	-	NSB 58L 97 P	-	1.0B 100L 100P
**HANT-AC**	1.0B 100L 100P	Reject3 p<0.0001	-	1.0B 96L 91P	-	1.0B 100L 100P
**nlsu**	1.0B 100L 100P	1.0B 96L 95P	1.0B 92L 88P	-	1.0B 100L 100P	-
**nssu**	1.0B 100L 100P	1.0B NSL NSP	1.0B 92L 92P	-	1.0B 100L 99P	-
**RPB2**	1.0B 100L 100P	1.0B 100L 99P	1.0B 100L 99P	-	1.0B 100L 98P	Reject p<0.0001
**RDNA**4**+RPB2**	1.0B 100P	1.0B 100P	1.0B 100P	-	1.0B 100P	-
**rDNA**	100L	81L	81L	-	100L	Reject p<0.05

1 Support values (B-Bayesian posterior probabilities, L-maximum likelihood bootstraps, P-maximum parsimony bootstraps.

2 No support from this method.

3 Shimodaira-Hasegawa test significance level p<0.05.

4 nssu+nlsu concatenated.

The phylogeny of the fHANT-AC genes in fungi is similar to the organismal phylogeny inferred with rRNA and RPB2 genes, with one striking exception: in independent and combined analyses, the three genes of the fHANT-AC of the ascomycete *T. reesei* were strongly supported (1.0 BPP, 100 MPB, 100 MLB) as sister to those of the basidiomycete *Ustilago maydis*. Additionally, a Shimodaira-Hasegawa test (Table 1) rejected the monophyly of ascomycete nitrate assimilation clusters when *T. reesei* sequences were included (p<0.0001). Together, these analyses indicate strong conflict between the organismal and nitrate assimilation gene phylogenies that is best explained by HGT of the fHANT-AC from the Ustilaginales to *Trichoderma*. A search of the EST database at GenBank confirms that at least two of the horizontally transferred genes (*nrt2* and *euknr*) are expressed in *T. reesei*. We recovered no evidence of vertically derived homologs of these genes in *T. reesei*

**Figure 2:**
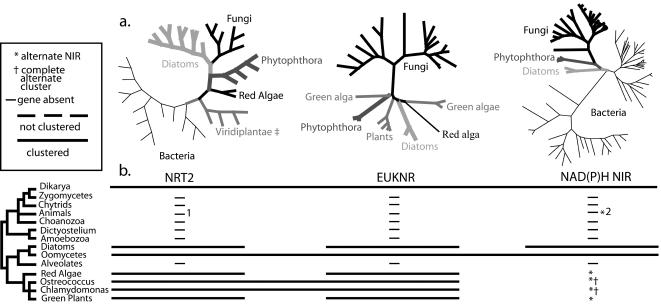
Distribution and phylogeny of HANT-AC gene homologs. (a) Unrooted phylograms of maximum likelihood analyses performed in RaxML-VI-HPC ver. 2.2.3. Support values for critical nodes can be found in Table 2. Contributing phylogenetic analyses are in SI Fig. 2a,b,c. (b) Distribution and clustering of HANT-AC genes in eukaryotes. Clustering indicated if most or all genomes in clade show clustering. Phylogeny of eukaryotes is adapted from Baldauf [57]. ^1^A homolog similar to bacterial sequences is present in *Nematostella vectensis.* ^2^ *N. vectensis* may also possesses bacteria-like nitrite reductase homologs.

Higher-level analyses (Fig. 2) of HANT-AC proteins including diverse eubacteria sequences along with sequences from fungi, heterokonts and 25-29 (depending on gene distribution) eukaryotic lineages are not inconsistent with a heterokont origin of the fungal HANT-AC, although patchy distribution prevents a strong conclusion from phylogenetic analyses. NAD(P)H-NIR sequences from three species of *Phytophthora* (oomycetes) and *Phaeodactylum* and *Thalassiosira* (diatoms) do not form a heterokont clade. *Phytophthora* NAD(P)H-NIR sequences are sister to fungal sequences (1.0 BPP, 81 MPB, 57 MLB), and heterokonts plus fungi are supported as monophyletic by MP analyses (77 MPB), but not by ML or Bayesian analyses. Analyses of NRT2 suggest a red alga+heterokont+fungi clade (1.0 BPP, <50 MPB, 74 MLB) that excludes green algae and plants in a strongly supported (1.0 BPP, 92 MPB, 99 MLB) eukaryote clade. These results are consistent with previous analyses extensively focused on fungi, which suggested that fungal NRT2 sequences form a monophyletic group that is nested within a paraphyletic assemblage of sequences from heterokonts [5]. Analyses of EUKNR sequences , which were restricted to the eukaryotic distribution of the gene, revealed little phylogenetic resolution outside the fungi. We found no synapomorphic insertion or deletion events in any of these sequences that exclusively support a sister relationship between fungi and oomycetes. Future analyses that include eukaryotic and fungal lineages not represented in our survey could falsify the hypothesis of an ancient transfer from heterokonts to dikarya.

**Table 2:** Alignments including diverse eukaryotic sequences.

Dataset				Clade				
	**Fungi**	**Heterokonts**	**Asco-mycetes**	***T. reesei*** **+Basidiomycetes**	**Fungi+** ***Phytophthora***	**Fungi+ Heterokonts**	***Ustilago*** **+** *T. reesei*	**Chromalveolates+Plantae**
**NRT2**	1.0B, 100L, 100P1	1.0B, 100L, 80 P	NT2	NT	-	-	NT	Reject p = .038
**EUKNR**	1.0B, 100L, 99P	-	-	1.0B, 100L, 98P	-	-	1.0B, 100L, 100P	1.0B, 100L, 100P
**NAD(P)H NIR**	1.0B, 100L, 100P	Reject3 P<.0001	-	1.0B	1.0B, 97 L, 91P	84P	1.0B, 100L, 100P	NT
**5nuc. prot.** 4	1.0B, 100L, 100P	1.0B, 100L, 100P	NT	NT	Reject p<.0001	Reject p = .008	NT	1.0B, 100L, 100P

1 Support values (B-Bayesian posterior probabilities, L-maximum likelihood bootstraps, P-maximum parsimony bootstraps.

2 Clade not tested by this alignment.

3 Shimodaira-Hasegawa test significance level p<.05.

4 Five nuclear proteins (α-tubulin, β-tubulin, RPB1, RPB2 and EF1-α) were derived from genome projects as described in methods.

### Gene Clustering of fHANT-AC genes is Conserved, but Order is Not

While fHANT-AC genes are usually clustered, gene orders within and flanking the cluster are not well conserved between lineages (Fig. 1a). A comparison of genes flanking the fHANT-AC (Fig. 1b) in *Ustilago maydis* and *T. reesei* genomes did not reveal additional genes that appear to have been obtained by HGT. Most genes on the *T. reesei* scaffold 28 are highly similar to ascomycete genes (data not shown). In addition, the *Cip2* gene that occurs three genes downstream from the *nrt2* gene is a confirmed *T. reesei* sequence [18]. These results suggest that the incongruous fHANT-AC was incorporated by chromosomal recombination involving a small number of genes rather than by acquisition of an additional chromosome. Genes flanking the fHANT-AC in *T. reesei* appear to be related to carbohydrate metabolism. We speculate that the occurrence of this cluster could influence carbohydrate metabolism by either upregulating or inhibiting transcription of these genes, and thereby contribute downstream to the superlative propensity of *T. reesei* to produce cellulases, *e.g.*in [19].

## Discussion

### The fHANT-AC persists with genomic rearrangement

The only groups found to maintain the three genes of the fHANT-AC in a cluster (as of June 2007) are the dikarya fungi and oomycete heterokonts (SI Table S1). *Euknr* and *nrt2* are widely distributed in dikarya fungi, chromalveolates (alveolates+heterokonts), and plants, whereas *NAD(P)H-nir* does not occur outside heterokonts and Dikarya (Fig. 2). We cannot yet falsify the hypothesis that the fHANT-AC cluster originated in the lineage leading to heterokonts (SI Fig. S3A) and was subsequently transferred to Dikarya. Because of the limited sample of non-dikarya fungi (SI Table S1) we cannot rule out the possibility of an earlier fungal origin. Nitrate assimilation clusters also occur in at least two green algae [20], [21], but each of these clusters contains a unique complement of genes that are not all homologous to those in the fHANT-AC. The convergent evolution of a nitrate assimilation cluster in green algae could suggest a general selective advantage to clustering of nitrate assimilation genes.

Clustering of nitrate assimilation genes is observed in varying degrees within Dikarya (Fig. 1a). In Basidiomycota where the genes are present, the clustered orientation of the three genes predominates with one known exception. A retroviral sequence interrupts the cluster between *nrt2* and *NAD(P)H-nir* in *Laccaria bicolor*, but the genes remain in the same locus. The cluster has disassembled in certain ascomycetes. At least two lineages have lost the clustering of the three genes while maintaining the genes themselves, most notably the Sordariomycetes, of which *T. reesei* is a member. *Botryotinia fuckeliana* maintains a cluster of two of the genes, while the closely related *Sclerotinia sclerotiorum* lacks clustering entirely. The orientation of the genes within the cluster is not phylogenetically conserved within Dikarya, suggesting that while rearrangement is common, these rearrangements do not necessarily disassemble the cluster. We propose three scenarios to explain the disassembly of the fHANT-AC in ascomycetes (SI Fig. S3B, C, D). If cluster formation is assumed to be an unlikely event, then that would favor a scenario (SI Fig. S3D) that only requires the assembly of the fHANT-AC to have occurred once. Metabolic gene clusters have been reported in several fungal lineages [22], [23], [24], [25], [26], [27], and one occurrence of rapid assembly of a cluster has been suggested in yeast [28]. However, there is currently no consensus regarding methods of measuring the relative rates of cluster assembly, disassembly and horizontal transfer in fungi.

### The Anomalous fHANT-AC in *Trichoderma reesei* coincides with Niche Shift

*T. reesei,* an ascomycete adapted to wood decay occurs in a genus consisting largely of endophytes and parasites [29]. *T. reesei* strain QM9414, the subject of the *T. reesei* genome project, was derived from QM6a, a strain originally defined by its inability to grow on nitrate as a sole nitrogen source, likely due to defects in nitrite reductase [30]. Several sexually competent strains of *T. reesei,* however, are known to utilize nitrate [30]. We know of no direct evidence of nitrate assimilation by other *Trichoderma* species, although the presence of nitrate has been linked to physiological responses [31], [32], [33] and suppression of growth [34] in some species. Attempts to amplify *nrt2* from various *Trichoderma* species using PCR were not successful. A shift away from a tightly regulated nitrate assimilation pathway may have preceded the acquisition of a basidiomycete fHANT-AC by *T. reesei*. Analyses of gene synteny (Fig. 1a) suggest that loss of the ascomycete nitrate assimilation genes in *T. reesei* was preceded by the disassembly of the cluster in Sordariomycetes. In one study, *Trichoderma* species (not including *T. reesei*) and *Sclerotinia sclerotiorum* (which also lacks an intact cluster) were shown to utilize ammonium preferentially, and possibly exclusively (in *Trichoderma)*, over nitrate in ammonium nitrate feeding experiments [35]. The effect was also observed, but less pronounced, for other sordariomycetes (*Fusarium spp*.). These observations suggest that there was a change in the mode of nitrate assimilation in the evolution of sordariomycetes. This could have favored the acquisition of the basidiomycete fHANT-AC and subsequent loss of vertically inherited genes when *T. reesei* switched to a nitrogen-deprived, woody substrate. The horizontal transmission leading to the *T. reesei* cluster is a plausible scenario because some *Trichoderma* species are intracellular parasites of basidiomycetes [36], [37].

### Analysis of the HANT-AC Suggests Widespread Loss, Convergence or a “Selfish Operon”

The distribution of HANT-AC genes could suggest either widespread loss outside Dikarya, heterokonts and plants or an HGT origin of the fungal homologs. We can reject the null hypotheses (Table 2) that chromalveolate and plant NRT2 form a clade that excludes fungi (p = .038), that heterokont NAD(P)H-NIR are monophyletic exclusive of fungi (p<.0001), and that an organismal phylogeny based on five nuclear protein coding genes (SI Fig. S2d), restricted to taxa bearing HANT-AC genes, is consistent with the fungi+*Phytophthora* clade (p<.0001) suggested by NAD(P)H-NIR analyses. Each of these results is consistent with an HGT origin of these genes in fungi. The eukaryotic *NAD(P)H-nir* could have been derived from a mitochondrial source, which would require multiple losses in different lineages of eukaryotes, or there could have been a more recent transfer from bacteria to a lineage leading to the heterokonts. The absence of *euknr* from other opisthokonts also suggests that this gene was secondarily acquired in Dikarya, although phylogenetic analyses do not clearly identify a source. The existence in *Phytophthora* of a cluster (oHANT-AC) homologous to the fHANT-AC, some of whose components appear to be vertically derived, is the strongest evidence of a heterokont origin of the fungal genes, implying that a cluster could have facilitated HGT. We present an hypothesis of the origin of the fHANT-AC in SI Fig. S3, which parsimoniously accounts for the observed distribution of HANT-AC genes in eukaryotes. There we show a single origin of the cluster in heterokonts from genes present in common ancestors of heterokonts and plants, followed by a transfer to the ancestor of Dikarya.

Recent studies have suggested closer genetic links between oomycetes and fungi than would be predicted by organismal phylogeny. One suggested that a number of shared EST sequences relate to a mutual tendency toward pathogenicity [38]. It was not clear if this is evidence of genomic convergence or genetic exchange. Another study suggested that at least four genes have been transferred from ascomycete fungi to oomycetes [39]. A failure to detect homologous sequences in other heterokonts and non-dikarya fungi in that study leaves the origin of these sequences ambiguous, requiring the invocation of additional prokaryotic donors. In our study, the presence of homologous genes in other heterokonts and related lineages, but not in other fungi suggests that the direction of the hypothetical transfer of the fHANT-AC would be from a heterokont to a common ancestor of dikarya fungi. A limited sample of non-dikarya fungi leaves an earlier fungal origin also possible. In this case, the restriction of clustered HANT-AC genes to oomycetes suggests that the heterokonts could have produced the species that donated the fHANT-AC to dikarya fungi. While the current study alone is not conclusive as to genetic exchange between these groups, it contributes to a developing hypothesis of an ancient, intimate association involving Dikarya and heterokonts.

The alternate hypothesis is that the HANT-AC arose convergently in fungi and oomycetes under similar environmental pressures. For example, in aerobic soils, reduced iron for denitrification is limited, leading to elevated nitrate [40]. Iron-containing ferredoxin is required to donate electrons in plant nitrite reduction, but this function can be performed by NAD(P)H (derived from cellular respiration) in fungi and heterokonts; they could be partly freed from iron limitations through this mechanism. That certain diatoms are dominant in high-nitrate, low-iron environments [41] seems to support this. This nutritional mode could benefit photosynthetic partners in ectomycorrhizal and lichen symbioses under aerobic conditions. In fact, ectomycorrhizal fungi both down-regulate plant nitrite reductase [42] and increase iron uptake [43]. In contrast, oomycete plant pathogens could out-compete their host for nitrate in iron-limited conditions. Alternatively, the absence of photosystems to efficiently replenish reduced ferredoxin in fungi and oomycetes could have necessitated convergent acquisition of a respiration-linked source of electrons for nitrite reduction.

Co-regulation of the three genes in the fHANT-AC in *H. cylindrosporum* was recently demonstrated [3]. This was earlier demonstrated in the ascomycetous yeast, *Pichia angusta* [2]. The intergenic region between *NAD(P)H-nir* and *euknr* in *A. nidulans* serves as a bidirectional promoter for both genes [44], [45]. These findings collectively suggest that the fHANT-AC resembles a eukaryotic operon. The “selfish operon” is a coordinately regulated cluster of genes, which receive improved fitness because they are more readily maintained when transferred between organisms [8]. While not truly a “selfish” element due to its likely positive influence on host fitness, evidence of HGT of the fHANT-AC makes it a candidate for a “selfish” eukaryotic operon. The current results suggest that acquisition of nutritional operons may promote ecological shifts in fungi. Differential utilization of the fHANT-AC in various strains of *T. reesei* could suggest this species is currently undergoing such an evolutionary event.

### A key acquisition in colonization of dry land?

The Dikarya experienced a neoproterozoic diversification [46] that was not paralleled in other fungal lineages, including some that also associate with plants [17]. We speculate that the horizontally acquired ability to derive nitrogen from oxidized substrates, and to thus benefit photosynthetic hosts, may have been a key innovation that allowed the Dikarya to diversify in terrestrial habitats as free-living saprotrophs or mycorrhizal symbionts.

## Materials and Methods

### Phylogenetic Analysis of Organisms Containing the High Affinity Nitrate Assimilation Gene Cluster (fHANT-AC) using rRNA and RPB2 gene sequences

We used a combination of tblastn [47] and searches for annotated genes to obtain phylogenetically relevant sequences for species noted with an * in SI Table S1. Substitutions were made for *Pichia angusta* RNA polymerase II second largest subunit (RPB2, AAS67502, *Pichia guilliermondii*) and *Hebeloma cylindrosporum* (partial nuclear small ribosomal RNA subunit gene [nssu] AY745703 and *rpb2* DQ472718 from *H. velutipes* specimen PBM 2277) where genome projects were not available. We generated a partial nuclear large ribosomal RNA subunit gene (nlsu) sequence from *H.cylindrosporum* strain CBS558.96 (EF219136) using the primers LROR and LR5 (sequencing was performed on an ABI377 Automated DNA Sequencer using BigDye ver1.1; Applied Biosystems, Foster City, CA, USA). We concatenated nlsu and nssu nucleotide sequences and the inferred amino acid sequence of RPB2 for all available fungal genomes. Sequences were aligned with ClustalX [48], and adjusted manually. We created independent alignments (SI Table S2) for each gene by excluding two of the three genes, and a combined analysis that included all three genes. Ambiguously aligned characters were excluded from further analysis. Each alignment was analyzed by (A) 1000 maximum parsimony bootstrap replicates with ten random addition sequence replicates per bootstrap replicate, saving multiple trees at each replicate in PAUP* 4.0b [49], (B) two Bayesian analyses using mixed protein models for amino acids and a GTR plus gamma model of evolution for nucleotides in MrBayes ver. 3.2 [50], [51], [52]; we ran the Bayesian analyses for one million generations, sampled trees every 100 generations and removed trees sampled before likelihoods for two runs converged and stabilized as the burnin, and (C) 100 Maximum Likelihood Bootstrap replicates of RPB2 with mixed protein models and of combined rDNA with mixed GTR models as implemented in RaxML-VI-HPC ver. 2.2.3. We also performed (D) maximum likelihood searches of a combined rDNA dataset and an RPB2 dataset in RaxML-VI-HPC ver. 2.2.3 [53] (mixed GTR and protein models) for the optimal topologies and also for the best topology in which *T. reesei* sequences were constrained to form a clade with *U. maydis*. We then used a Shimodaira-Hasegawa test [54], as implemented in TreePuzzle ver. 5.2 [55], to compare the likelihoods of resulting topologies.

### Analysis of the nitrate assimilation gene cluster in fungi

We concatenated amino acid sequences inferred from three genes in the fHANT-AC (*nrt2, euknr* and *NAD(P)H-nir*) of *Hebeloma cylindrosporum*. We used tBlastn [47] with this sequence to search genome projects shown in supporting materials (SI Table S1) for homologous sequences. We obtained *H. cylindrosporum* and *P. angusta* fHANT-AC sequences, and *T. reesei* EST sequences from GenBank. The identity of each sequence was verified by a blastp search of GenBank to determine the most similar sequences and conservation of functional domain architecture. We assembled an alignment consisting of concatenated amino acid sequences from the fHANT-AC for all available fungal genomes (as of May, 2006, shown with an * in SI Table S1) and also *Phytophthora sojae* and *Phytophthora ramorum* genomes, which were included as the outgroup. Alignments and analyses for independent genes and combined sequences were conducted as described for amino acid sequences in the phylogenetic analysis. We performed maximum likelihood searches of the combined fHANT-AC dataset using mixed protein models in RaxML-VI-HPC (see above) for the optimal topology and also for the best topology in which ascomycete sequences were constrained to be monophyletic. We then compared the likelihoods of resulting topologies using a Shimodaira-Hasegawa test as described above for RPB2.

### Analyses of components of the fHANT-AC in diverse eukaryotes and prokaryotes

We conducted higher level analyses of NRT2, EUKNR and NAD(P)H-NIR amino acid sequences retrieved from eukaryotic genome projects along with bacterial sequences from GenBank. For NRT2, approximately 200 bacterial sequences and for NAD(P)H-NIR approximately 500 bacterial sequences were initially obtained. These were combined with the eukaryotic genome sequences, aligned with mafft ver. 5.861 [56] using the default settings. Long ends of the alignment were trimmed and a neighbor joining analysis was performed in PAUP* 4.0b. Thirty-nine clades of NRT2 and eighty-three clades of NAD(P)H-NIR were then analyzed as described above for the fHANT-AC genes, except 500 maximum likelihood bootstraps were performed on each gene separately. We confirmed that major clades from previous NRT2 analyses [5] were represented. Twenty-nine eukaryotic EUKNR sequences were also analyzed as described for the other protein sequences. Constraint analyses as described above forced the monophyly of heterokont NAD(P)H-NIR sequences. We performed analyses as described for a combined dataset of α-tubulin, β-tubulin, RNA polymerase II largest and second largest subunits, and elongation factor 1-alpha limited to taxa bearing homologs of genes in the fHANT-AC. We then performed constraint analyses as described above, which forced either fungi and *Phytophthora* or fungi and heterokonts to be monophyletic.

### Analysis of gene order and location in fungi

We used the browser at the *T. reesei* and *Ustilago maydis* genome websites to catalog genes flanking the fHANT-AC in each species. We cataloged genes of up to four inferred or hypothetical proteins for each species. We used tBlastn [47] with a concatenated amino acid sequence of the three proteins encoded by the fHANT-AC of *Coprinopsis cinerea* in order of nitrate metabolism (NRT2-EUKNR-NAD(P)H-NIR) to obtain the location and relative direction of transcription of each gene in the eukaryotic genomes and bacterial genomes shown in supporting materials, Table S1. We also performed tBlastn using the individual genes, and used 45% amino acid similarity to determine the absence of homologs for each gene in eukaryotes and 40% amino acid similarity in bacteria. This was repeated using the *Arabidopsis thaliana* ferredoxin-dependent nitrite reductase (NP_179164.1) as the query.

## Supporting Information

Figure S1 Figure S1 depicts phylogenetic trees of fungal sequences, rooted with an oomycete (heterokont) outgroup. Figure S1a shows phylogenetic trees based on independent and combined analyses of ribosomal RNA and RPB2 gene loci, which reflect the organismal phylogeny. Figure S1b presents phylogenetic trees based on independent and combined analyses of genes in the fHANT-AC cluster. All analyses of the organismal phylogeny group Trichoderma reesei with Gibberella zeae (ascomycetes), and all analyses of genes in the fHANT-AC cluster group Trichoderma reesei with Ustilago maydis (basidiomycetes).
(0.49 MB PDF)

Figure S2 Maximum likelihood trees showing relationships among (a) NRT2, (b) EUKNR and (c) NAD(P)H NIR amino acid sequences from eukaryotic and eubacteria genomes. (d) Maximum likelihood trees showing relationships among concatenated nuclear gene products (α- and β-tubulin, rpb1, rpb2 and ef1-α), from eukaryotic genomes. Branch lengths reflect number of changes per site.
(0.88 MB PDF)

Figure S3 Evolution of the nitrate assimilation cluster in Fungi and other eukaryotes. A. Evolution of the nitrate assimilation cluster across the eukaryote phylogeny. B. Cluster evolution within the fungi. C, D. Alternative reconstructions of cluster dissociation and formation events in fungi. 1. Nrt2 and NAD(P)H nir were acquired from bacteria, and euknr was derived from the sulfite oxidase gene family in the nuclear genome[1]. 2. The common ancestor of the Chromalveolates and Plantae retained nrt2, NAD(P)H-nir, and euknr. 3. NAD(P)H-nir was lost in the lineage leading to Plantae, after the divergence of Chromalveolates, and Cp-nir was acquired during the primary endosymbiotic origin of the chloroplast (not necessarily in that order). 4. Cp-nir was horizontally transferred from the Rhodophytes to the Heterokonts (Stramenopiles) during a secondary endosymbiotic origin of chloroplasts [2]. Cp-nir was retained in the lineage leading to the diatoms, but was lost in the lineage leading to the Oomycetes. 5. Nrt2, NAD(P)H-nir, and euknr were each lost in the lineage leading to the Alveolates. 6. The nitrate assimilation cluster, including nrt2, NAD(P)H-nir, and euknr, was formed in the lineage leading to the Oomycetes. 7. The nitrate assimilation cluster was horizontally transferred from the lineage leading to the Oomycetes to the lineage leading to Dikarya. 8. The nitrate assimilation cluster was horizontally transferred from the lineage leading to Ustilago maydis (Basidiomycota) to the lineage leading to Trichoderma reesei (Ascomycota). The Ascomycota-derived components of the nitrate assimilation cluster were lost in the lineage leading to Trichoderma reesei (this could have occurred before or after the horizontal transfer event). 9. The nitrate assimilation cluster became dissociated twice during the evolution of Ascomycota. Aspergillus retains an ancestral clustered condition. Two component genes of the nitrate assimilation cluster (euk-nr and NAD(P)H-nir) were rejoined in the lineage leading to Botryotinia fuckeliana. 10. Alternatively (B), the nitrate assimilation cluster became dissociated only once in the evolution of Ascomycota, and was reconstituted in the lineage leading to Aspergillus; euk-nr and NAD(P)H-nir were rejoined in the lineage leading to Botryotinia fuckeliana. 11. Another possible scenario (D) suggests that the nitrate assimilation cluster became dissociated in four separate events in the Ascomycota, and that the clustered condition in Aspergillus spp. and Botryotinia fuckeliana are both plesiomorphic. Although this scenario involves the same number of breakpoint/association events as those described in (B) and (C), it does not require that clusters of genes be reconstituted after having been disrupted. 12. All three component genes of the nitrate assimilation cluster were lost in the lineages leading to Cryptococcus neoformans (Basidiomycota) and Saccharomyces cerevisiae (Ascomycota). Vertical order of branching points does not reflect absolute timing of such events. 1. Stolz JF, Basu P (2002) Evolution of nitrate reductase: molecular and structural variations on a common function. Chembiochem 3: 198–206. 2. Bhattacharya D, Yoon HS, Hackett JD (2004) Photosynthetic eukaryotes unite: endosymbiosis connects the dots. Bioessays 26: 50–60.
(0.93 MB PDF)

Table S1 ^1^ Highest blastp sequence in GenBank, 88% similar to AAQ21342, uncultured bacterium nitrite reductase. ^2^ Highest tblastn hit in GenBank feature in acc. #CP000082 Psychrobacter arcticus 273-4, nitrate transporter. ^3^ Tblastn 62%similar to Arabidopsis thaliana GenBank acc. # NM_127123, nitrite reductase, 42% similar to feature in NT_165926, Aspergillus terreus sulfite reductase, beta subunit. ^4^ Tblastn 69% similar to 489AA of C.cinerea NAD(P)Hnir. ^5^ Locus ZP_00980374, nitrite/sulfite reductase, Tblastn 44% similar to Arabidopsis thaliana GenBank acc. #NP_179164.
(0.11 MB DOC)

Table S2 For each dataset, two simultaneous Bayesian analyses were run for one million generations, using MrBayes ver. 3.1.2 [1], [2]. Amino acid data was analyzed under mixed protein models, and nucleotide data under a GTR+Gamma model. Trees were sampled every 100 generations. Trees sampled before likelihood convergence and stabilization of independent chains (usually 500–2000) were removed as the burnin. Each alignment in SI Table 2 was also analyzed by maximum parsimony bootstrapping and maximum likelihood bootstrapping. In parsimony analyses, performed in PAUP*4.0b [3], characters were equally weighted. 1000 bootstrap replicates were performed. Each bootstrap replicate performed a heuristic search with 10 random addition sequence replicates, with the multitrees option turned on, using the TBR branch-swapping algorithm. Likelihood analyses were performed under mixed protein models or mixed GTR (for nucleotides). 100 bootstraps were performed on fungal alignments (Fig. S1), and 500 on eukaryotic alignments (Fig. S2) in RaxML-VI-HPC ver. 2.2.3[4]. Literature Cited: 1. Huelsenbeck JP, Ronquist F (2001) MRBAYES: Bayesian inference of phylogenetic trees. Bioinformatics 17: 754–755. 2. Ronquist F, Huelsenbeck JP (2003) MrBayes 3: Bayesian phylogenetic inference under mixed models. Bioinformatics 19: 1572–1574. 3. Swofford DL (2003) PAUP*. Phylogenetic Analysis Using Parsimony (*and Other Methods). Version 4. Sinauer Associates, Sunderland, Massachusetts. 4. Stamatakis A (2006) RAxML-VI-HPC: maximum likelihood-based phylogenetic analyses with thousands of taxa and mixed models. Bioinformatics 22: 2688–2690.
(0.03 MB DOC)
