# Supplementary material for: Horizontal Transfer of a Nitrate Assimilation Gene Cluster and Ecological Transitions in Fungi: A Phylogenetic Study
Source: PLoS One. 2007 Oct 31;2(10):e1097. doi: 10.1371/journal.pone.0001097 (PMC2040219; doi:10.1371/journal.pone.0001097)

Figure S1a

Phylogenetic analyses of organisms (BPP,MLB,MPB)

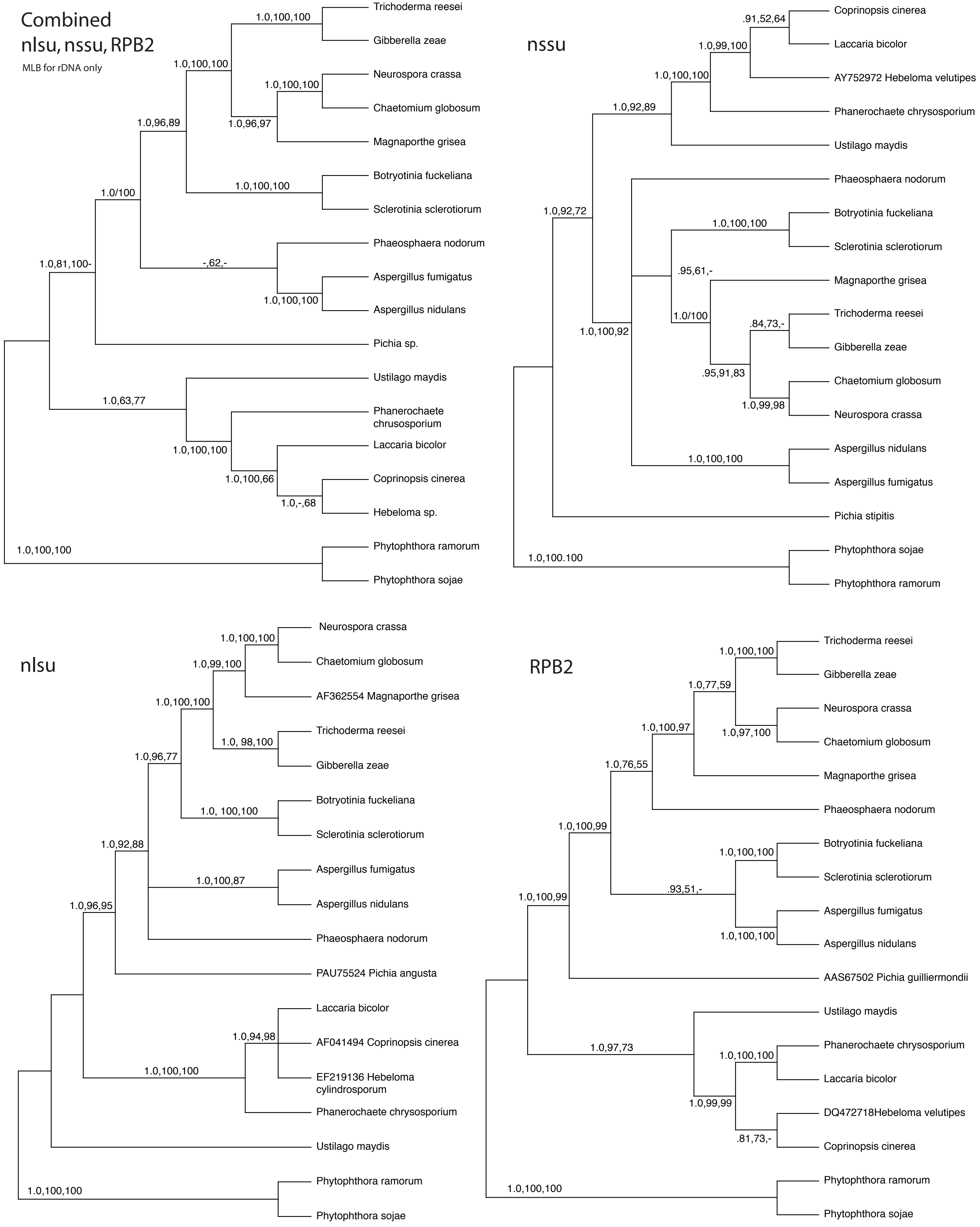

Figure S1b.

HANT-AC analyses (BPP,MLB,MPB)

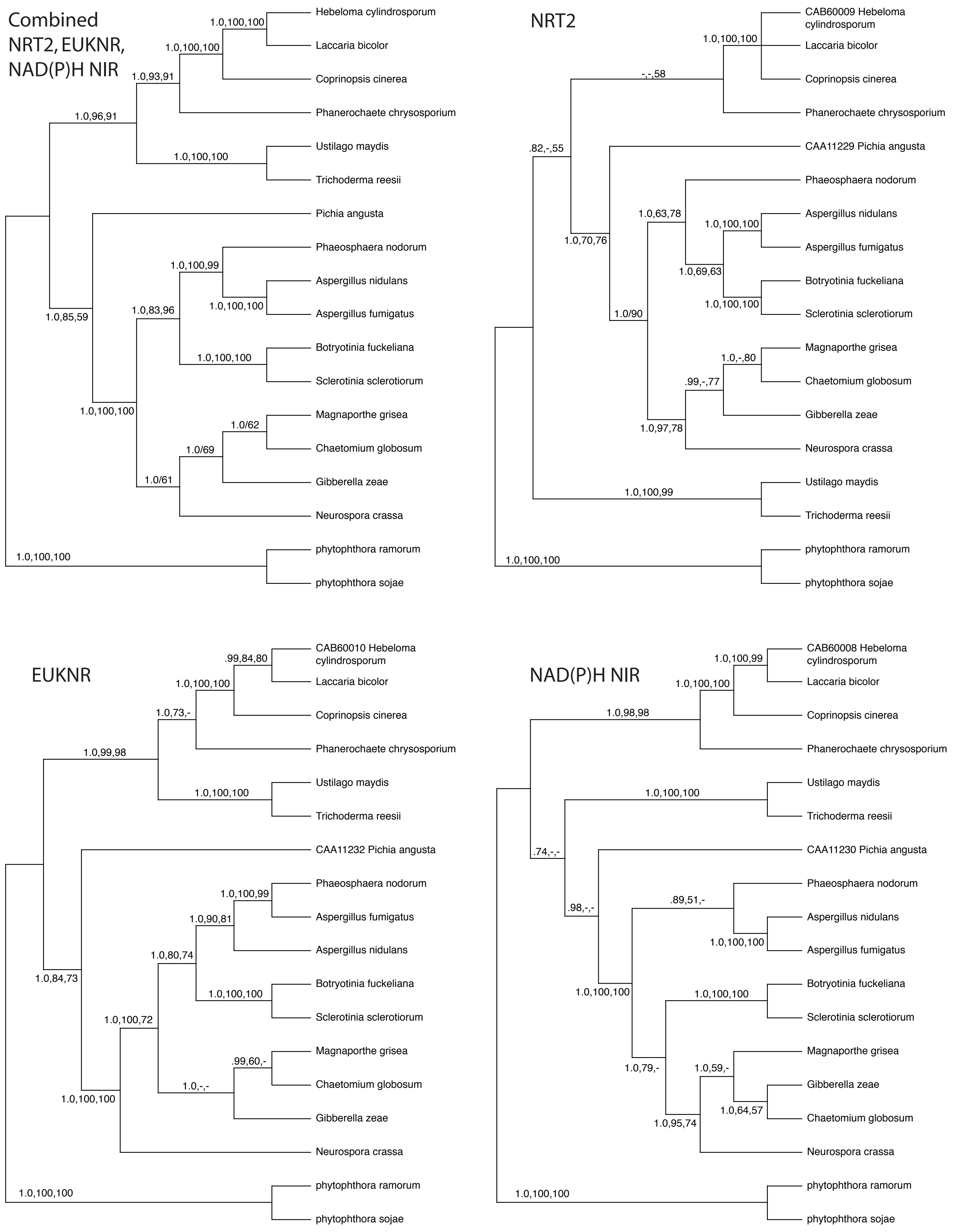

Supplement: Figure S1 — depicts phylogenetic trees of fungal sequences, rooted with an oomycete (heterokont) outgroup. Figure S1a shows phylogenetic trees based on independent and combined analyses of ribosomal RNA and RPB2 gene loci, which reflect the organismal phylogeny. Figure S1b presents phylogenetic trees based on independent and combined analyses of genes in the fHANT-AC cluster. All analyses of the organismal phylogeny group Trichoderma reesei with Gibberella zeae (ascomycetes), and all analyses of genes in the fHANT-AC cluster group Trichoderma reesei with Ustilago maydis (basidiomycetes). (0.49 MB PDF) [file pone.0001097.s001.pdf]
