# Supplementary material for: Horizontal Transfer of a Nitrate Assimilation Gene Cluster and Ecological Transitions in Fungi: A Phylogenetic Study
Source: PLoS One. 2007 Oct 31;2(10):e1097. doi: 10.1371/journal.pone.0001097 (PMC2040219; doi:10.1371/journal.pone.0001097)

Figure S2a
NRT2 (BPP,MLB,MPB)

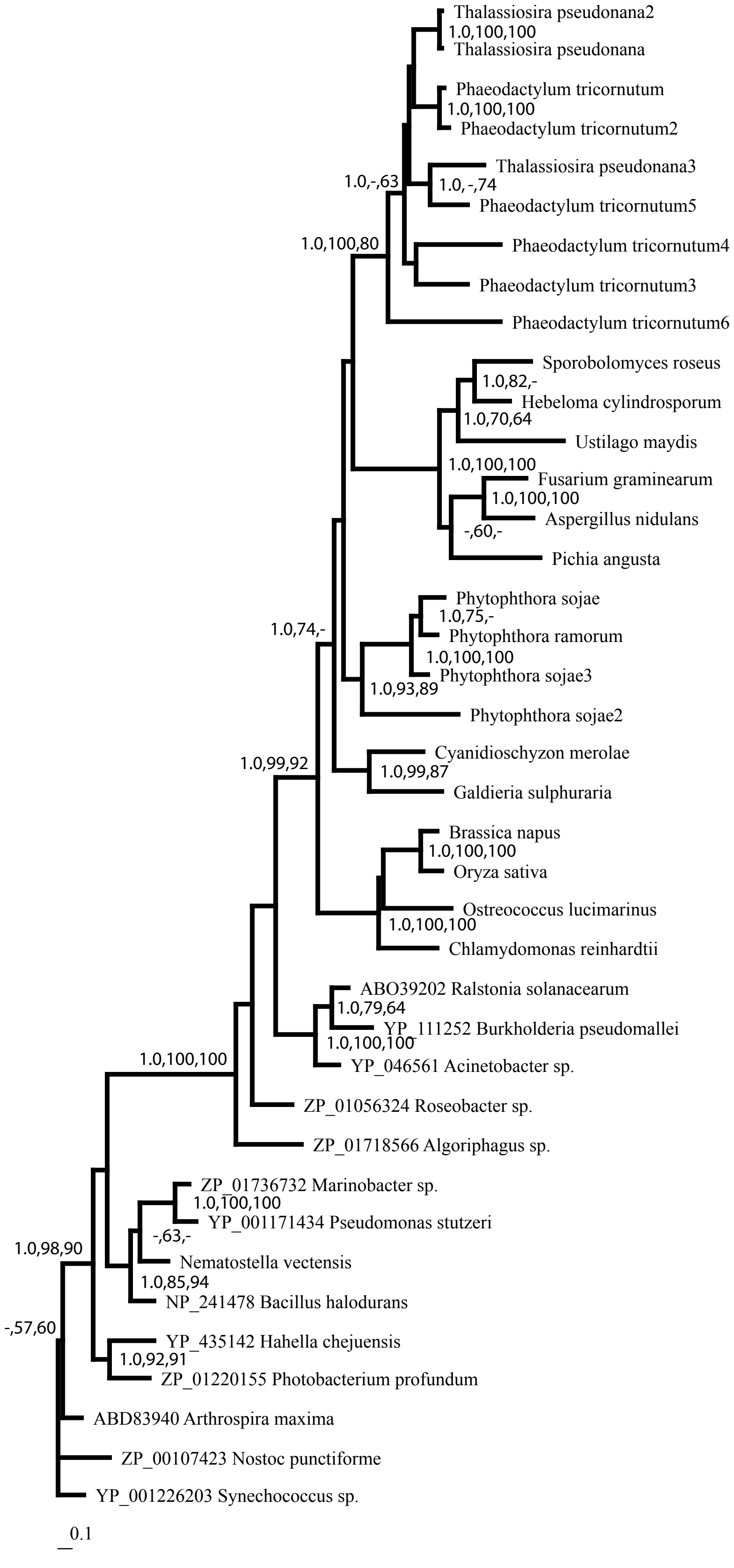

Figure S2b
EUKNR (BPP,MLB,MPB)

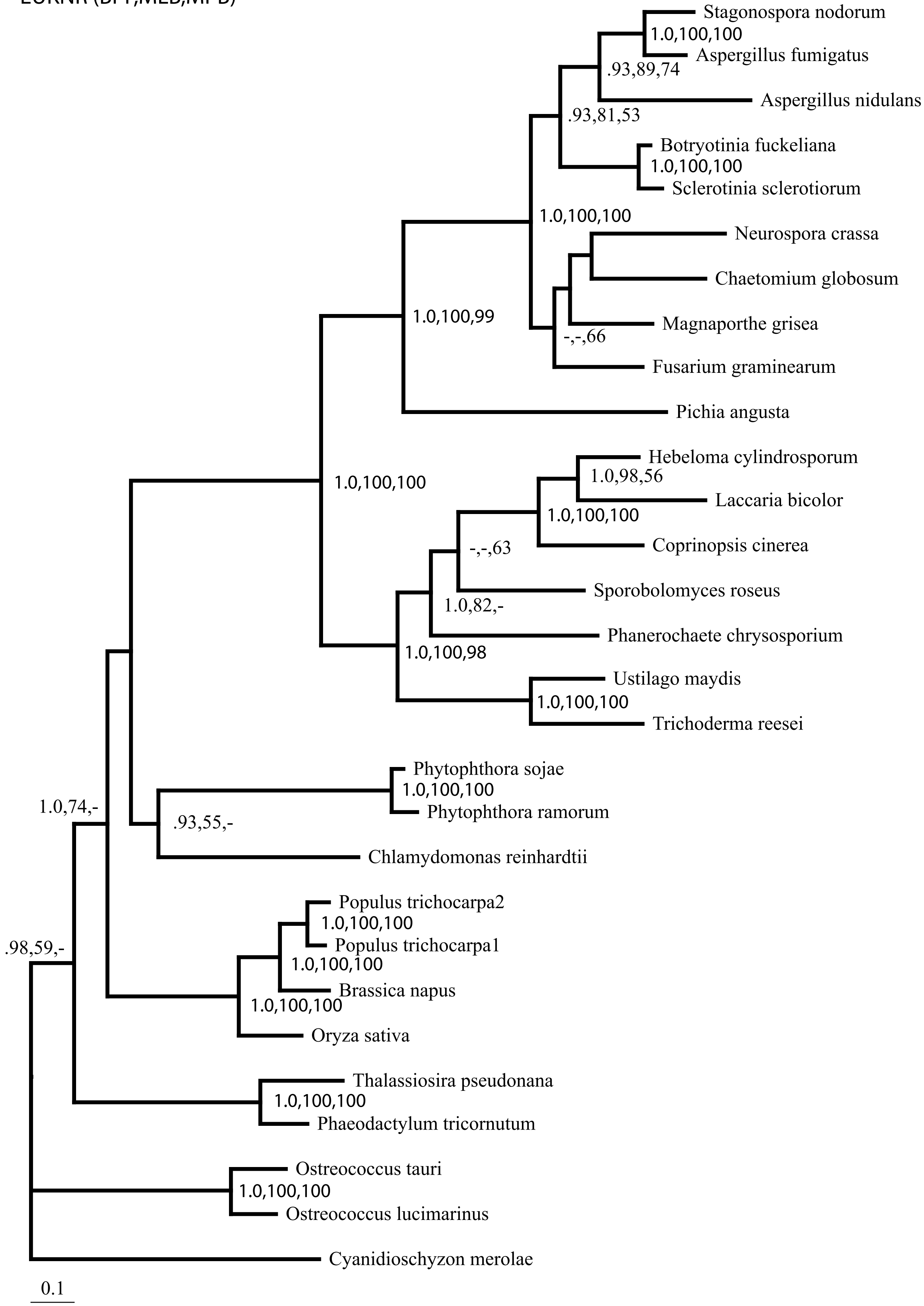

Figure S2c
NAD(P)H NIR (BPP<MLB<MPB)

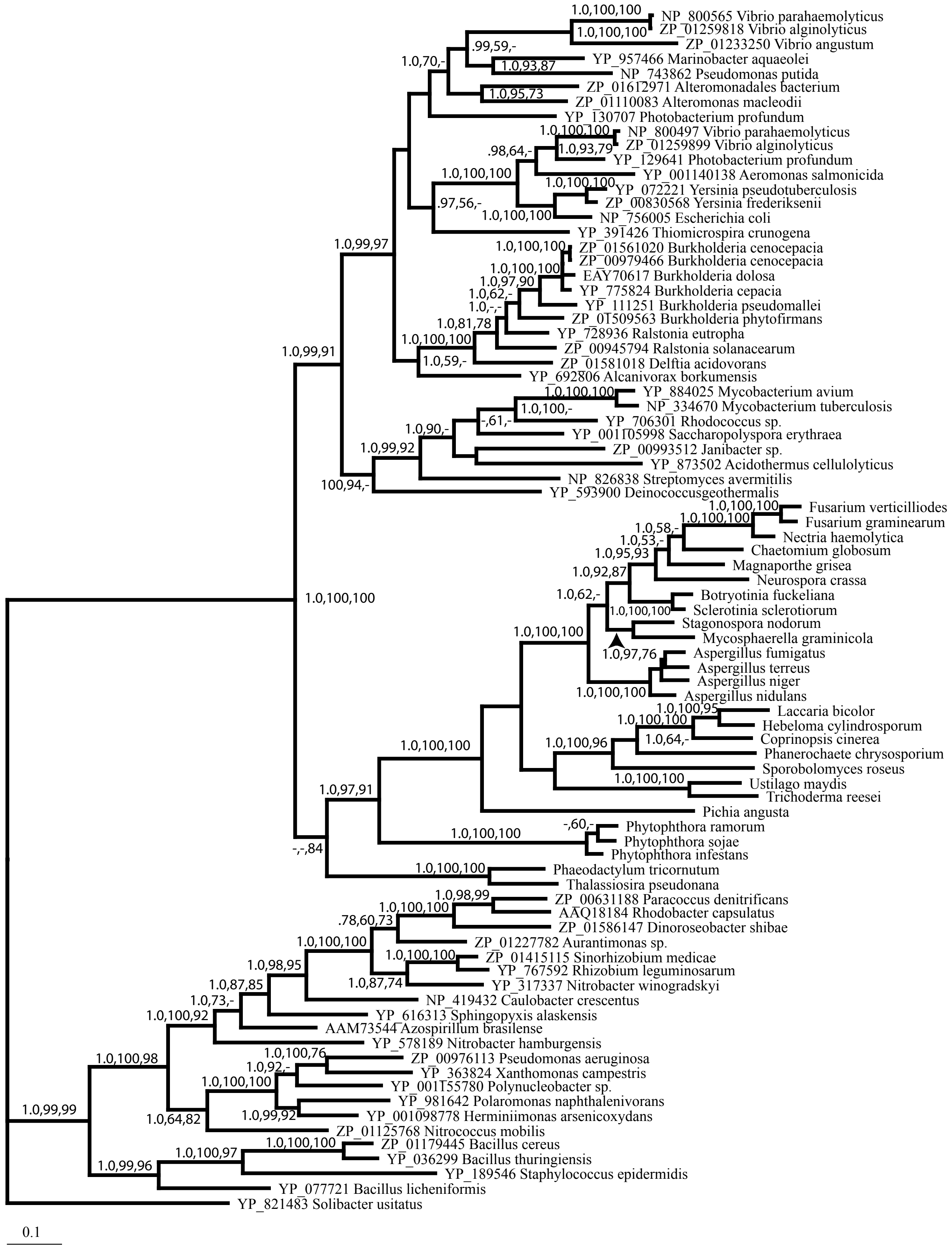

Figure S2d
Alpha-tubulin, beta-tubulin, RPB1, RPB2, EF1alpha AA (BPP,MLB,MPB)

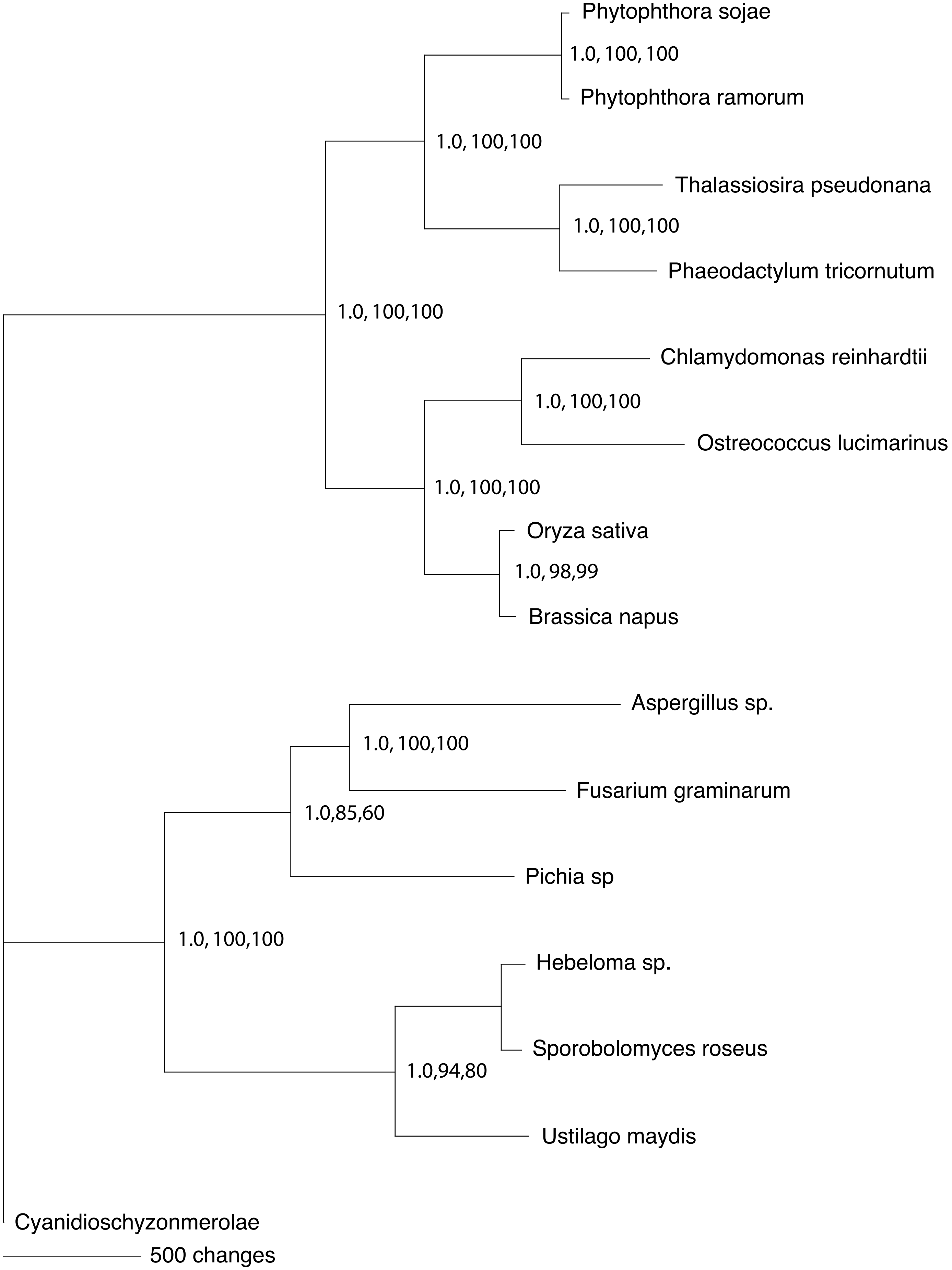

Supplement: Figure S2 — Maximum likelihood trees showing relationships among (a) NRT2, (b) EUKNR and (c) NAD(P)H NIR amino acid sequences from eukaryotic and eubacteria genomes. (d) Maximum likelihood trees showing relationships among concatenated nuclear gene products (α- and β-tubulin, rpb1, rpb2 and ef1-α), from eukaryotic genomes. Branch lengths reflect number of changes per site. (0.88 MB PDF) [file pone.0001097.s002.pdf]
