# Supplementary figures and images for: Horizontal Transfer of a Nitrate Assimilation Gene Cluster and Ecological Transitions in Fungi: A Phylogenetic Study
Source: PLoS One. 2007 Oct 31;2(10):e1097. doi: 10.1371/journal.pone.0001097 (PMC2040219; doi:10.1371/journal.pone.0001097)

Figure S3

**A**

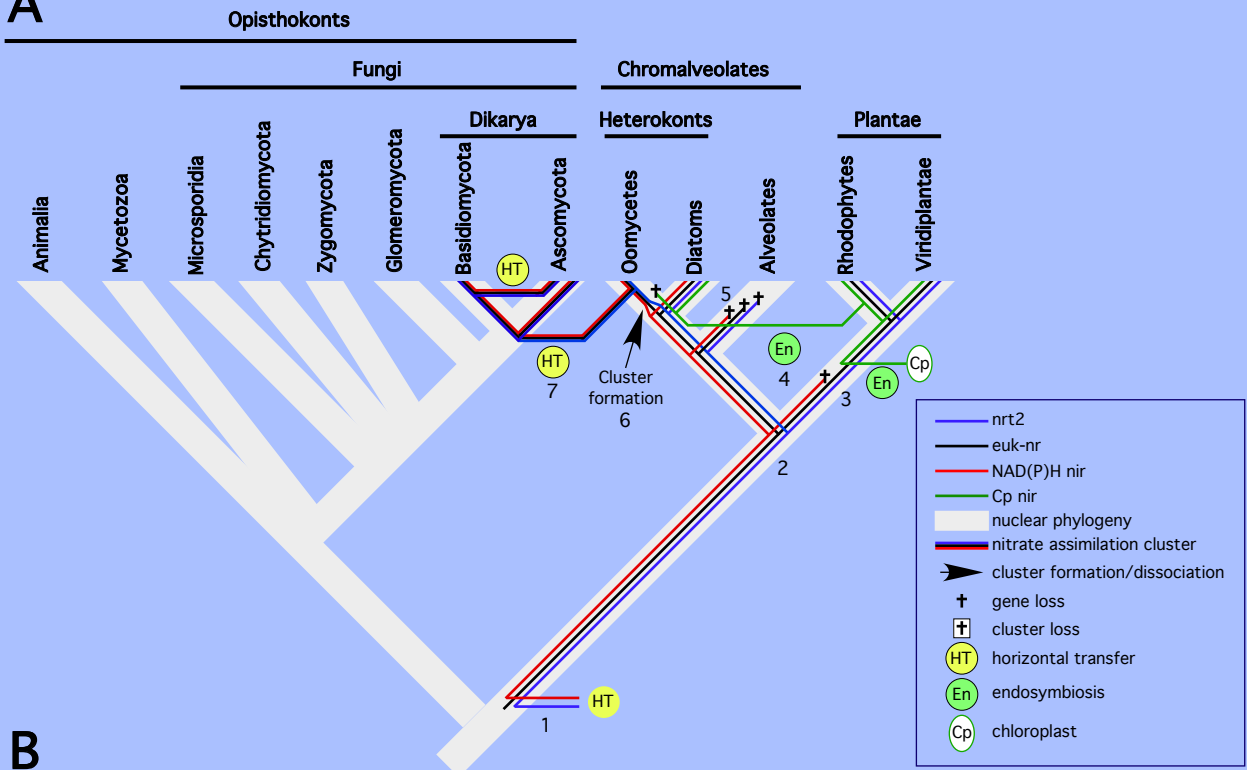

**B**

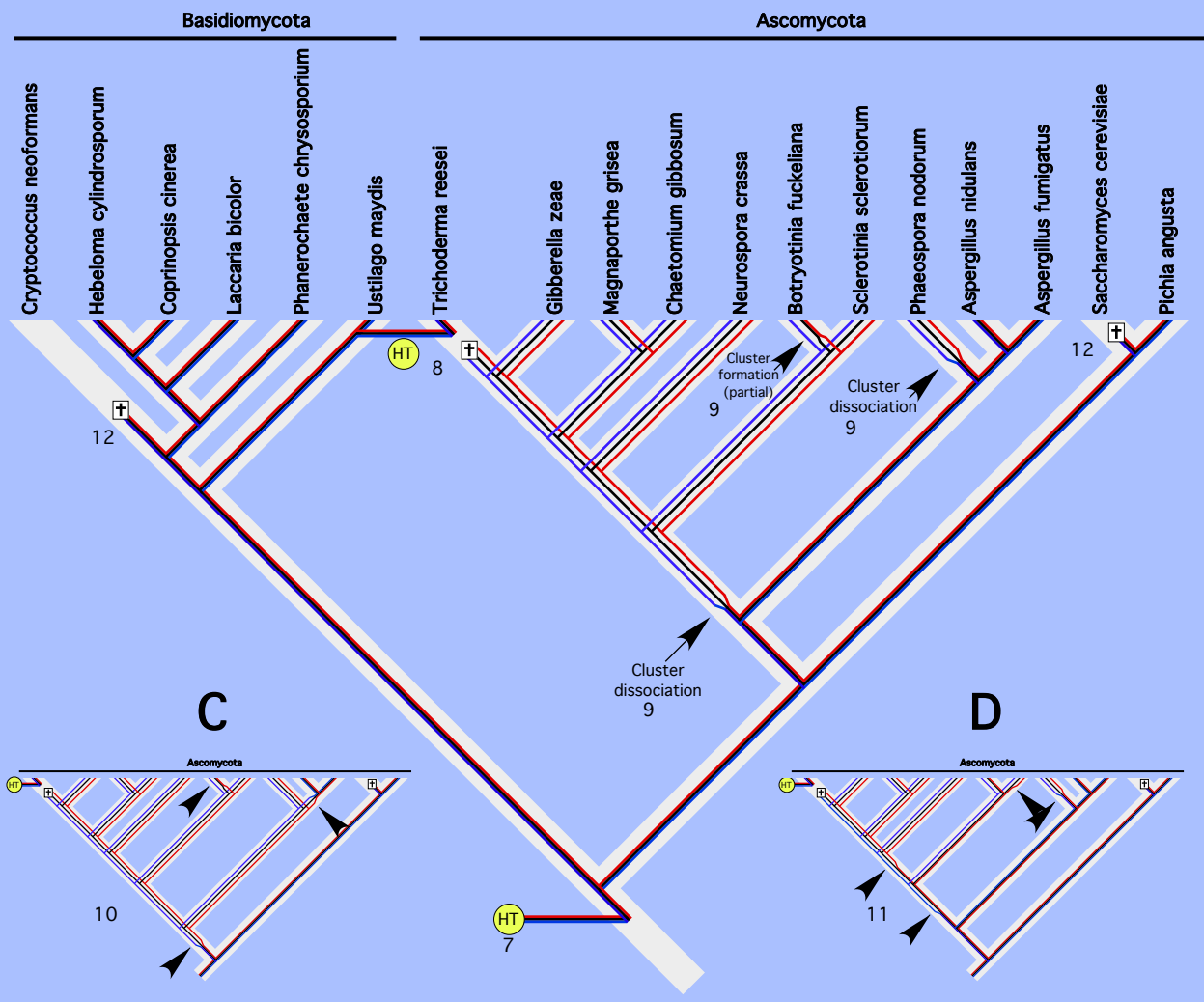

Supplement: Figure S3 — Evolution of the nitrate assimilation cluster in Fungi and other eukaryotes. A. Evolution of the nitrate assimilation cluster across the eukaryote phylogeny. B. Cluster evolution within the fungi. C, D. Alternative reconstructions of cluster dissociation and formation events in fungi. 1. Nrt2 and NAD(P)H nir were acquired from bacteria, and euknr was derived from the sulfite oxidase gene family in the nuclear genome[1]. 2. The common ancestor of the Chromalveolates and Plantae retained nrt2, NAD(P)H-nir, and euknr. 3. NAD(P)H-nir was lost in the lineage leading to Plantae, after the divergence of Chromalveolates, and Cp-nir was acquired during the primary endosymbiotic origin of the chloroplast (not necessarily in that order). 4. Cp-nir was horizontally transferred from the Rhodophytes to the Heterokonts (Stramenopiles) during a secondary endosymbiotic origin of chloroplasts [2]. Cp-nir was retained in the lineage leading to the diatoms, but was lost in the lineage leading to the Oomycetes. 5. Nrt2, NAD(P)H-nir, and euknr were each lost in the lineage leading to the Alveolates. 6. The nitrate assimilation cluster, including nrt2, NAD(P)H-nir, and euknr, was formed in the lineage leading to the Oomycetes. 7. The nitrate assimilation cluster was horizontally transferred from the lineage leading to the Oomycetes to the lineage leading to Dikarya. 8. The nitrate assimilation cluster was horizontally transferred from the lineage leading to Ustilago maydis (Basidiomycota) to the lineage leading to Trichoderma reesei (Ascomycota). The Ascomycota-derived components of the nitrate assimilation cluster were lost in the lineage leading to Trichoderma reesei (this could have occurred before or after the horizontal transfer event). 9. The nitrate assimilation cluster became dissociated twice during the evolution of Ascomycota. Aspergillus retains an ancestral clustered condition. Two component genes of the nitrate assimilation cluster (euk-nr and NAD(P)H-nir) were rejoined in th [file pone.0001097.s003.pdf]
