# Supplementary material for: Horizontal Transfer of a Nitrate Assimilation Gene Cluster and Ecological Transitions in Fungi: A Phylogenetic Study
Source: PLoS One. 2007 Oct 31;2(10):e1097. doi: 10.1371/journal.pone.0001097 (PMC2040219; doi:10.1371/journal.pone.0001097)
Supplement: Table S1 — 1 Highest blastp sequence in GenBank, 88% similar to AAQ21342, uncultured bacterium nitrite reductase. 2 Highest tblastn hit in GenBank feature in acc. #CP000082 Psychrobacter arcticus 273-4, nitrate transporter. 3 Tblastn 62%similar to Arabidopsis thaliana GenBank acc. # NM_127123, nitrite reductase, 42% similar to feature in NT_165926, Aspergillus terreus sulfite reductase, beta subunit. 4 Tblastn 69% similar to 489AA of C.cinerea NAD(P)Hnir. 5 Locus ZP_00980374, nitrite/sulfite reductase, Tblastn 44% similar to Arabidopsis thaliana GenBank acc. #NP_179164. (0.11 MB DOC) [file pone.0001097.s004.doc]

Table S1: Distribution of high affinity nitrate assimilation genes.

| Genome project | URL | ecology | genes |
| --- | --- | --- | --- |
| Rhodophyta |  |  |  |
| *Galdieria sulphuraria* | http://genomics.msu.edu/galdieria/ | autotroph | nrt2 euknr cpnir |
| *Cyanidioschyzon merolae* | http://merolae.biol.s.u-tokyo.ac.jp/ | autotroph | nrt2 euknr |
| Viridiplantae |  |  |  |
| *Arabidopsis thaliana* | http://www.tigr.org/tdb/e2k1/ath1/ | autotroph | nrt2 euknr cpnir |
| *Zea mays* | http://compbio.dfci.harvard.edu/tgi/cgi-bin/tgi/Blast/index.cgi | autotroph | nrt2 euknr cpnir |
| *Medicago trunculata* | http://compbio.dfci.harvard.edu/tgi/cgi-bin/tgi/Blast/index.cgi | autotroph | nrt2 euknr cpnir |
| *Triticum aestivium* | http://www.tigr.org/tdb/e2k1/tae1/ | autotroph | nrt2 euknr cpnir |
| *Oryza sativa* | http://www.tigr.org/tdb/e2k1/tae1/ | autotroph | nrt2 euknr cpnir |
| *Populus trichocarpa v1.1* | http://genome.jgi-psf.org/Poptr1/Poptr1.home.html | autotroph ectomycorrhizal | nrt2 euknr NADPHnir^1^ cpnir |
| *Chlamydomonas reinhadtii* | http://genome.jgi-psf.org/Chlre3/Chlre3.home.html | autotroph | nrt2 euknr cpnir |
| *Ostreococcus lucimarinus v2.0* | http://genome.jgi-psf.org/Ost9901_3/Ost9901_3.home.html | autotroph | nrt2 euknr cpnir |
| *Ostreococcus tauri v2.0* | http://genome.jgi-psf.org/Ostta4/Ostta4.home.html | autotroph | nrt2 euknr cpnir |
| Heterokonts |  |  |  |
| **Phytophthora sojae v1.1* | http://genome.jgi-psf.org/sojae1/sojae1.home.html | plant pathogen | 4nrt2 euknr NADPHnir |
| **Phytophthora ramorum v1.1* | http://genome.jgi-psf.org/ramorum1/ramorum1.home.html | plant pathogen | 3nrt2 euknr NADPHnir |
| *Thalassiosira pseudonana v3.0* | http://genome.jgi-psf.org/thaps1/thaps1.home.html | autotroph | nrt2 euknr NADPHnir cpnir |
| *Phaeodactylum tricornutum v2.0* | http://genome.jgi-psf.org/cgi-bin/runAlignment?db=Phatr2&advanced=1 | autotroph | nrt2 euknr NADPHnir cpnir |
| Metazoa |  |  |  |
| *Homo sapiens* | http://www.ensembl.org/Homo_sapiens/index.html | heterotroph |  |
| *Nematostella vectensis* | http://genome.jgi-psf.org/Nemve1/Nemve1.home.html | heterotroph | nrt2^2^ |
| *Xenopus tropicalis v4.1* | http://genome.jgi-psf.org/Xentr4/Xentr4.home.html | heterotroph |  |
| *Ciona intestinalis v2.0* | http://genome.jgi-psf.org/Cioin2/Cioin2.home.html | animal parasite |  |
| *Fugu rubripes v4.0* | http://genome.jgi-psf.org/Takru4/Takru4.home.html | heterotroph |  |
| Microsporidia |  |  |  |
| *Encephalitozoon cuniculi* | http://www.cns.fr/externe/English/Projets/Projet_AD/AD.html | intracellular parasite |  |
| Fungi *(sample of projects searched)* |  |  |  |
| *Batrachochytrium_dendrobatidis* | http://www.broad.mit.edu/annotation/genome/batrachochytrium_dendrobatidis | animal pathogen |  |
| *Cryptococcus neoformans* | http://www.broad.mit.edu/annotation/genome/cryptococcus_neoformans/Home.html | animal symbiont |  |
| *Phycomyces blakesleeanus* | http://genome.jgi-psf.org/Phybl1/Phybl1.home.html | heterotroph saprotroph |  |
| *Rhizopus oryzae* | http://www.broad.mit.edu/annotation/genome/rhizopus_oryzae/Home.html | heterotroph saprotroph |  |
| *Glomus (EST)* | http://darwin.nmsu.edu/~fungi/ | endomycorrhizal |  |
| *Candida lusitanian* | http://www.broad.mit.edu/annotation/genome/candida_lusitaniae/Home.html | animal symbiont |  |
| *Coccidioides immitis* | http://www.broad.mit.edu/annotation/genome/coccidioides_immitis/Home.html | animal symbiont |  |
| *Pichia stipitis v2.0* | http://genome.jgi-psf.org/Picst3/Picst3.home.html | methylotrophic heterotroph |  |
| *Aspergillus niger v1.0* | http://genome.jgi-psf.org/Aspni1/Aspni1.home.html | heterotroph saprotroph | nrt2 euknr NADPHnir |
| **Laccaria bicolor v1.0* | http://genome.jgi-psf.org/Lacbi1/Lacbi1.home.html | ectomycorrhizal | nrt2 euknr NADPHnir |
| *Nectria haematococca v1.0* | http://genome.jgi-psf.org/Necha1/Necha1.home.html | plant pathogen | nrt2 euknr NADPHnir |
| **Phanerochaete chrysosporium v2.0* | http://genome.jgi-psf.org/Phchr1/Phchr1.home.html | heterotroph saprotroph | nrt2 euknr NADPHnir |
| **Trichoderma reesei v1.0* | http://gsphere.lanl.gov/trire1/trire1.home.html | heterotroph saprotroph (fungal/ plant parasite?) | nrt2 euknr NADPHnir |
| *Coprinopsis cinerea* | http://www.broad.mit.edu/annotation/genome/coprinus_cinereus/Home.html | heterotroph saprotroph | nrt2 euknr NADPHnir |
| *Ustilago maydis* | http://www.broad.mit.edu/annotation/genome/ustilago_maydis/Home.html | plant pathogen | nrt2 euknr NADPHnir |
| *Sporobolomyces roseus* | http://genome.jgi-psf.org/cgi-bin/runAlignment?db=Sporo1&advanced=1 | plant pathogen | nrt2 euknr NADPHnir |
| *Gibberella zeae* | http://www.broad.mit.edu/annotation/genome/fusarium_graminearum/Home.html | plant pathogen | nrt2 euknr NADPHnir |
| *Magnaporthe grisea* | http://www.broad.mit.edu/annotation/genome/magnaporthe_grisea/Home.html | plant pathogen | nrt2 euknr NADPHnir |
| *Chaetomium globosum* | http://www.broad.mit.edu/annotation/genome/chaetomium_globosum/Home.html | plant pathogen | nrt2 euknr NADPHnir |
| *Neurospora crassa* | http://www.broad.mit.edu/annotation/genome/neurospora/Home.html | heterotroph saprotroph | nrt2 euknr NADPHnir |
| *Botryotinia fuckeliana* | http://www.broad.mit.edu/annotation/genome/botrytis_cinerea/Home.html | plant pathogen | nrt2 euknr NADPHnir |
| *Sclerotinia sclerotiorum* | http://www.broad.mit.edu/annotation/genome/sclerotinia_sclerotiorum/Home.html | plant pathogen | nrt2 euknr NADPHnir |
| *Phaeosphaera nodorum* | http://www.broad.mit.edu/annotation/genome/stagonospora_nodorum/Home.html | plant pathogen | nrt2 euknr NADPHnir |
| *Aspergillus nidulans* | http://www.broad.mit.edu/annotation/genome/aspergillus_nidulans/Home.html | heterotroph saprotroph | nrt2 euknr NADPHnir |
| *Aspergillus fumigatus* | http://www.sanger.ac.uk/Projects/A_fumigatus/ | animal pathogen | nrt2 euknr NADPHnir |
| Alveolata |  |  |  |
| *Tetrahymena thermophila* | http://www.tigr.org/tdb/e2k1/ttg/ | heterotroph |  |
| *Plasmodium falciparum* | http://www.tigr.org/tdb/e2k1/pfa1/ | animal parasite |  |
| Choanoflagellate |  |  |  |
| *Monosiga brevicollis* | http://genome.jgi-psf.org/Monbr1/Monbr1.home.html | heterotroph |  |
| Amoeboflagellate |  |  |  |
| *Naegleria gruberi v1.0* | http://genome.jgi-psf.org/Naegr1/Naegr1.home.html |  |  |
| Bacteria |  |  |  |
| *Synechococcus WH8102* | http://genome.jgi-psf.org/finished_microbes/synw8/synw8.home.html | phototroph | Nrt2 cpnir^3^ |
| *Burkholderia cenocepacia pc184* | http://www.broad.mit.edu/annotation/genome/burkholderia_cenocepacia/Home.html | endobacterium | Nrt2 NAD(P)Hnir^4^ cpnir^5^ |
