# Supplementary material for: Horizontal Transfer of a Nitrate Assimilation Gene Cluster and Ecological Transitions in Fungi: A Phylogenetic Study
Source: PLoS One. 2007 Oct 31;2(10):e1097. doi: 10.1371/journal.pone.0001097 (PMC2040219; doi:10.1371/journal.pone.0001097)
Supplement: Table S2 — For each dataset, two simultaneous Bayesian analyses were run for one million generations, using MrBayes ver. 3.1.2 [1], [2]. Amino acid data was analyzed under mixed protein models, and nucleotide data under a GTR+Gamma model. Trees were sampled every 100 generations. Trees sampled before likelihood convergence and stabilization of independent chains (usually 500–2000) were removed as the burnin. Each alignment in SI Table 2 was also analyzed by maximum parsimony bootstrapping and maximum likelihood bootstrapping. In parsimony analyses, performed in PAUP*4.0b [3], characters were equally weighted. 1000 bootstrap replicates were performed. Each bootstrap replicate performed a heuristic search with 10 random addition sequence replicates, with the multitrees option turned on, using the TBR branch-swapping algorithm. Likelihood analyses were performed under mixed protein models or mixed GTR (for nucleotides). 100 bootstraps were performed on fungal alignments (Fig. S1), and 500 on eukaryotic alignments (Fig. S2) in RaxML-VI-HPC ver. 2.2.3[4]. Literature Cited: 1. Huelsenbeck JP, Ronquist F (2001) MRBAYES: Bayesian inference of phylogenetic trees. Bioinformatics 17: 754–755. 2. Ronquist F, Huelsenbeck JP (2003) MrBayes 3: Bayesian phylogenetic inference under mixed models. Bioinformatics 19: 1572–1574. 3. Swofford DL (2003) PAUP*. Phylogenetic Analysis Using Parsimony (*and Other Methods). Version 4. Sinauer Associates, Sunderland, Massachusetts. 4. Stamatakis A (2006) RAxML-VI-HPC: maximum likelihood-based phylogenetic analyses with thousands of taxa and mixed models. Bioinformatics 22: 2688–2690. (0.03 MB DOC) [file pone.0001097.s005.doc]

Table S2: Description of alignments analyzed in this study.

| Figure | Alignment | Included characters (#) | Parsimony informative characters (#) |
| --- | --- | --- | --- |
| S1a | Combined nssu, nlsu, RPB2 | 4301, 3637 (rDNA likelihood) | 1283 |
| S1a | nssu | 1824 | 374 |
| S1a | nlsu | 1813 | 403 |
| S1a | RPB2 | 1085 | 534 |
| S1b | fHANT-AC combined | 2381 | 1651 |
| S1b | NRT2 (fungal) | 468 | 386 |
| S1b | EUKNR(fungal) | 851 | 597 |
| S1b | NAD(P)H NIR (fungal) | 1062 | 668 |
| S2a | NRT2 (eukaryote) | 424 | 325 |
| S2b | EUKNR (eukaryote) | 785 | 547 |
| S2c | NAD(P)H NIR (eukaryote) | 1082 | 855 |
